# Being in a “Green” Building Elicits “Greener” Recycling, but Not Necessarily “Better” Recycling

**DOI:** 10.1371/journal.pone.0145737

**Published:** 2016-01-05

**Authors:** David W.-L. Wu, Alessandra DiGiacomo, Peter J. Lenkic, Vanessa K. Wong, Alan Kingstone

**Affiliations:** Department of Psychology, University of British Columbia, 2136 West Mall, Vancouver, British Columbia, V6T 1Z4, Canada; Tulane University Health Sciences Center, UNITED STATES

## Abstract

Previous observational work revealed that transient populations in a sustainable building disposed of waste more accurately when compared to patrons in a non-sustainable building. The current study uses an experimental design to replicate this observed effect and to investigate whether or not the built environment influences motivational factors to impact behavior. We find support that a building designed and built to communicate an atmosphere of sustainability can influence waste disposal behavior. Participants in the sustainable building used the garbage receptacle significantly less and compensated by tending to select the containers and organics receptacle more, which actually resulted in more errors overall. Our findings suggest that building atmospherics can motivate people to recycle more. However, atmospherics alone do not appear to be sufficient to elicit the desired performance outcome.

## Introduction

Psychology research is being applied increasingly to situations that involve pro-environmental behaviors (see e.g. [1], introducing a special section in *European Psychologist* on the topic). The hope is that such research will not only increase our understanding of human behavior, decision-making, and motivation (for a review, see [2]), but also ultimately inform policy decisions to combat the environmental degradation problems that humanity currently faces [3].

Psychology researchers have studied numerous pro-environmental behaviors, including: energy conservation [4, 5], green purchases [6], sustainable eating [7], littering [8], and recycling [9]. Recycling is the most studied pro-environmental behavior and the focus in the literature has been to promote such behavior by enacting specific interventions [10]. These interventions range from manipulating convenience [11] to the use of information cues [12]. The effectiveness of such interventions are often considered from the theory of planned behavior (see e.g. [13]). This theory suggests that three main variables–one’s attitude toward the behavior, subjective norms, and perceived behavioral control–influence one’s intention and thus the behavior itself [14].

However, the theory of planned behavior does not emphasize how one’s physical context might impact behavior. Even more recent theoretical frameworks that combine the theory of planned behavior with other popular theories of environmental behavior also fail to include physical context as a major variable [15]. Nevertheless while some see such a factor outside of the theory of planned behavior [16], physical context might be relevant if it impacts the convenience, and therefore the perceived difficulty (an aspect of perceived behavioral control) of the behavior [17]. Indeed, the effectiveness of recycling interventions may well depend on the context within which the behavior occurs [18]. Yet to date the influence of contextual factors on recycling, and pro-environmental behaviors more broadly, has not been studied systematically [19].

Contextual factors, such as the built environment one is in, can have strong effects on one’s cognition, attitude, and ultimately, on one's behaviour. This not only includes specific attributes of the built environment, like the amount of natural light [20], but also the design of the space as a whole. In the built environment and marketing literature, the term “atmospherics” is used and defined as the “intentional control and structuring of environmental cues” [21, 22]. Building atmospherics can influence consumer behavior [21], educational outcomes [23], health outcomes [24], and workplace productivity [25]. In the context of “green” buildings, there has been evidence of enhanced user experience and productivity [26, 27], but little on how green buildings can impact pro-environmental behavior.

One of our previous studies was, to our knowledge, the first to investigate how simply being in a green building might influence recycling behaviour on a transient population [28]. We observed that patrons publically disposing waste in a cafeteria were almost 30% more likely to sort out their waste correctly in a sustainable building compared to patrons in a non-sustainable building, despite no difference between the buildings in population demographics or explicit environmentally-related motivations for choosing the cafeteria. This finding demonstrates that the same category of behavior occurring in two locations can dramatically differ in outcome, even in a transient population of users. We proposed and considered that the different atmospherics of the buildings might explain the results, but because of the study's observational design we could not directly test the idea.

How might the built environment affect recycling behavior? Steg and Vlek [19] proposed that contextual factors might influence behavior in one of four ways. First, it can do so directly. For example, one could physically remove or add options. To wit, one would not expect recycling behavior if only garbage bins are available to patrons.

The second way that contextual factors might alter behavior is indirectly by influencing factors, like attitudes, affect, and norms, that change one’s motivation [19]. Steg and Vlek [19] give the example that the introduction of recycling facilities may promote more positive attitudes towards recycling. Of particular relevance to the present study, the use of norms has been effective in promoting behaviors that are relatively easy to do but with few perceived benefits (low barrier, low reward behaviors). Providing normative information (e.g. how many other people engage in the behavior) has been shown to have robust effects on energy conservation behaviors [18]. Schultz [29] found that providing residents with average recycling rates of the community was effective in increasing the frequency of recycling and the amount of material recycled. Further, a social norm in an office environment that promotes a commitment to recycle has been found to directly influence office recycling behavior [30]. The implication is that the atmospherics of a building designed with sustainability in mind may influence what one perceives to be the social norm in the building, thus altering behavior. For example, highly visible or creative recycling facilities may promote norms relating to waste disposal in a building [31].

Thirdly, individual differences might modulate contextual effects and their influence on environmental behavior. For example, Steg and Vlek [19] give the example of recycling facilities promoting recycling only among those who score high in environmental concern. Lastly, contextual factors may change one’s “goal-frame”. That is, the presence of recycling facilities can encourage a normative goal-frame (“to act appropriately”) rather than a hedonic goal frame (“to feel better right now”).

The purpose of the present study is to follow-up on our observational findings in Wu et al. [28]–that individuals in a sustainable building are more likely to sort waste correctly than in a non-sustainable building–by using an experimental design to determine 1) whether the main findings of our observational study replicate in an experimentally controlled setting, and 2) to begin to identify how the built environment might alter waste disposal behavior between these two building environments.

Our focus was on Steg and Vlek [19]’s second mechanism: the effects of the building on motivational factors like norms. This mechanism of action may be especially important given Schultz [18]’s prediction that motivational factors should be the most effective method to increase low barrier, low reward behaviors like public recycling [10]. By using an experimental design, we could control and equate the physical arrangement of the disposal infrastructure. Random assignment ensured that the personal factors of the participants would not differentially influence our results, and giving all participants the same task would control their goal-frame. Thus, any difference in performance would be attributed to the building influencing motivational factors.

However, it should be noted that just as there are limits in an observational study, so too are there limitations inherent in employing an experimental design. Participants act differently, often pro-socially, just by virtue of the fact that they know they are in an experimental situation [32]. Therefore, participants in the present study may use the compost bins more readily, or spend more time at the bins as an overt pro-social signal, compared to patrons in the observational study. A supplementary component of the present study was to assess this possibility.

## Methods

All participants provided written consent to participate in this study. The consent procedure and study was done with ethical approval from the University of British Columbia Behavioural Research Ethics Board.

### Participants

Fifty-seven students from the University of British Columbia were given course credit or paid $5 to participate in the study. Participants were randomly assigned to be tested in either the sustainable building or the non-sustainable building. Five participants were removed from the sustainable building condition and eight participants were removed from the non-sustainable building condition because of failure to dispose of any items, failure to record usable video, or participants predicting the aim of the study (all participants were asked during debriefing what they thought the purpose of the study was). This left 22 participants in the sustainable building condition (7 males, 15 females, average age = 23.8), and 22 participants in the non-sustainable building condition (9 males, 13 females, average age = 21.5).

### Materials

**Fig 1:**
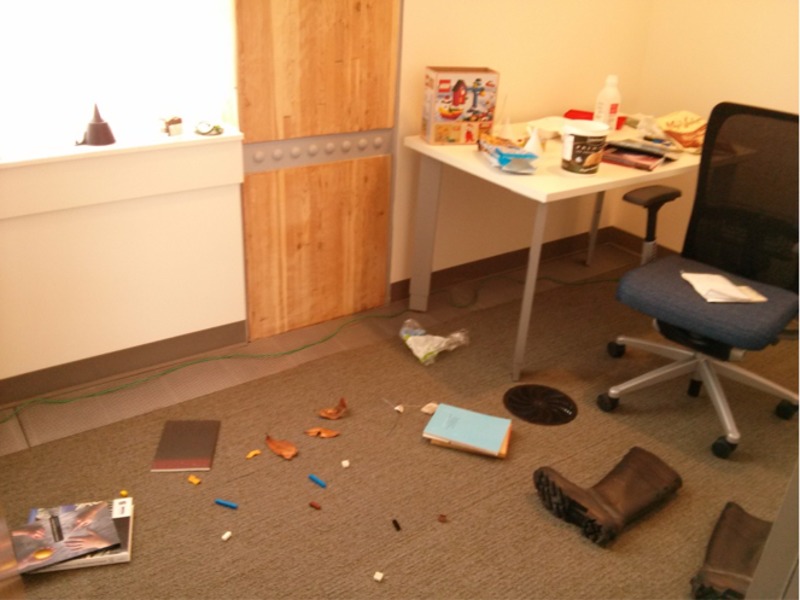
The room that participants were instructed to tidy up.

**Fig 2:**
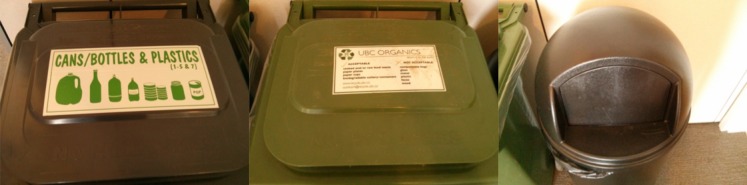
The three categories of receptacles made available to the participants. From left to right: containers, organics, and garbage.

Office rooms similar in size and features were used in either the sustainable building or the non-sustainable building. The room was created to look messy as illustrated in Fig 1. In the room, there were six items that were designated containers, organics, or garbage items (Table 1) for a total of 18 test items. Aside from the test items, there were other items to be organized as well, such as textbooks, boots, a cardigan etc., to convince participants of the organization task. The arrangement of the items in the room was consistent between buildings, with items being placed in logical locations in the rooms (e.g. spoons were placed in food containers, leaves were placed on the ground). A container, organics, and garbage receptacle was located in the hallway next to the door into the office room (Fig 2). These receptacles are used throughout the university campus. Participants wore a pair of glasses with a video recorder (720P HD Camera Eyewear) during the experimental task as a deception to draw participants away from the true purpose of the study. A hidden Logitech webcam was used to film the participants at the receptacles.

**Table 1:** List of items in each waste category.

Containers	Organics	Garbage
Beer bottle	Tea bag	Plastic wrap
Beer can	Paper towel	Broken pen
Perrier bottle	Orange peel	Plastic fork
Juice box	Dry pasta	Plastic muffin bag
Juice bottle	Paper take-out container	Lollipop wrapper
Plastic take-out container	Dry leaves	Coffee cup lid

### Procedure

**Fig 3:**
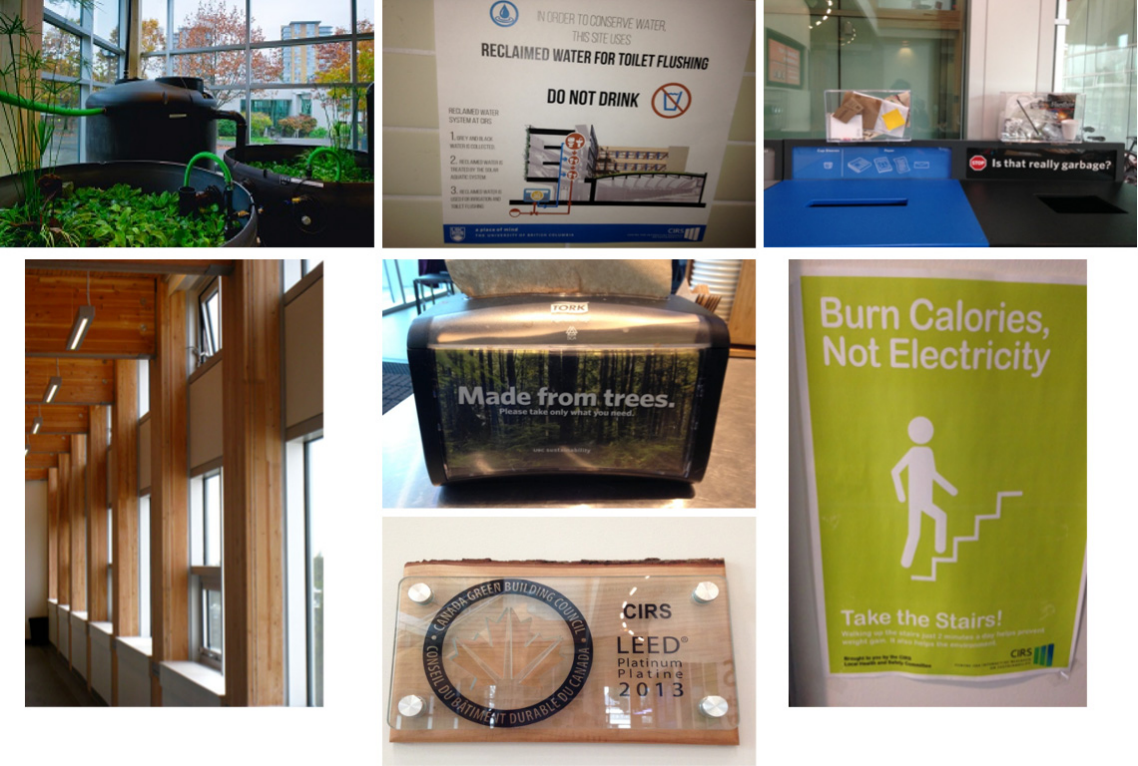
Examples of how the CIRS building conveys an atmosphere of sustainability to users. From top left to bottom right: water collection and purification system, signage in washroom about the water collection and purification system, 3D signage at waste receptacles in the café, extensive use of wood in the building’s construction, messaging on paper napkin holders in the café, LEED platinum certification display in the lobby, signage by the elevators to increase the use of stairs.

Participants in the sustainable building condition were tested in the Centre for Interactive Research on Sustainability (CIRS). This certified LEED Platinum and award-winning regenerative building was designed and built with one of its core aims being to encourage sustainable practices by actively and intentionally embodying a message of sustainability. The building is designed as a teaching tool, employing a vast array of interventions to encourage occupants to engage in sustainability issues [31]. Notwithstanding “sustainability” in the name of the building and the certification displayed in the lobby area, an atmosphere of sustainability is created through the infrastructure of the building itself (e.g. an external “green wall” made of ivy, a rooftop garden, a prominent water collection and purification system by the main entrance, photovoltaic cells in the skylight glass, use of natural light and wood materials, etc.). In addition, messaging throughout the building, including prompts on napkin-holders and in washrooms, contribute to the atmosphere of sustainability (see Fig 3). The cafeteria serves healthy meals with locally sourced ingredients–different from other food venues across campus–and uniquely most things are compostable, including the wooden cutlery. UBC’s Sustainability Education Resource Centre is also housed in the lobby area. These design features help visitors visualize sustainability, and are not only educational, but also aspire to fascinate and inspire visitors [31, 33]. Previous data show that visitors in this building feel more environmentally conscious than those in other buildings [28]. Participants in the non-sustainably-designed building were tested in the Douglas Kenny Building which is made primarily out of concrete, has little natural light, only ventilated air, and is in almost all ways the anti-thesis of the CIRS building. While the Douglas Kenny Building is primarily composed of faculty, staff, and psychology students, it would be a mistake to think that the population of the two buildings is entirely different, as approximately 20% of the psychology faculty are housed in CIRS, plus the CIRS lecture theatre is used for psychology classes.

Participants were tested individually and went directly to the building they were assigned. They were told in the study's description that the investigation was about attention while organizing items in a real-world setting (the participants signed-up for the study online through the Brain and Attention Research Lab, which primarily does attention experiments). The purpose was reiterated by the experimenter when the participants arrived. As part of the cover story, participants were given glasses to wear that had a small camera on them to track their head motion. The experimenter walked the participant around the building putatively to familiarize the participants with the glasses, though the real purpose was to ensure that participants were exposed to the “feel” (i.e., the atmospherics) of the building. After touring the building, the participant was led into the experimental room, outside the eyes of the building occupants. They were asked to organize the messy room in 15 minutes and were told they could dispose of anything in the receptacles located just outside the room. Participants were unaware they were being filmed when they were at the receptacles, and were unaware that the glasses were never turned on.

### Analysis

The video footage was used to analyze how often receptacles were used and the amount of time spent at the receptacles. We were interested in the amount of time spent at the receptacles as it appeared that participants were very careful in disposing of their items, often spending much time reading through all the signs on the receptacles. This appeared to be a significant departure from what we saw in our initial observational study where people were disposing their food related items after eating in a cafeteria setting [28].

Our primary interest was to examine how accuracy differed across building conditions. After each participant completed his or her task, the experimenter examined the receptacles to determine the contents of each bin. While there are several ways to determine accuracy, the simplest is to calculate what percentage of the six items that belonged in each receptacle actually ended up in that receptacle. We also provide the global bin contents so readers can see the bigger picture of where each category of item tended to end up.

## Results

### Receptacles used

Videos were coded to determine which receptacles were selected in the two buildings. Coding was done by two research assistants, one of whom was blind to the study. Coding reliability was high, as evidenced by Cronbach’s alpha (containers: α = 0.95, organics = α = 0.95, and garbage: α = 0.97). It is important to note that not all participants disposed of all the 18 items, thus the average number of selections for each bin do not add up to 18.

**Fig 4:**
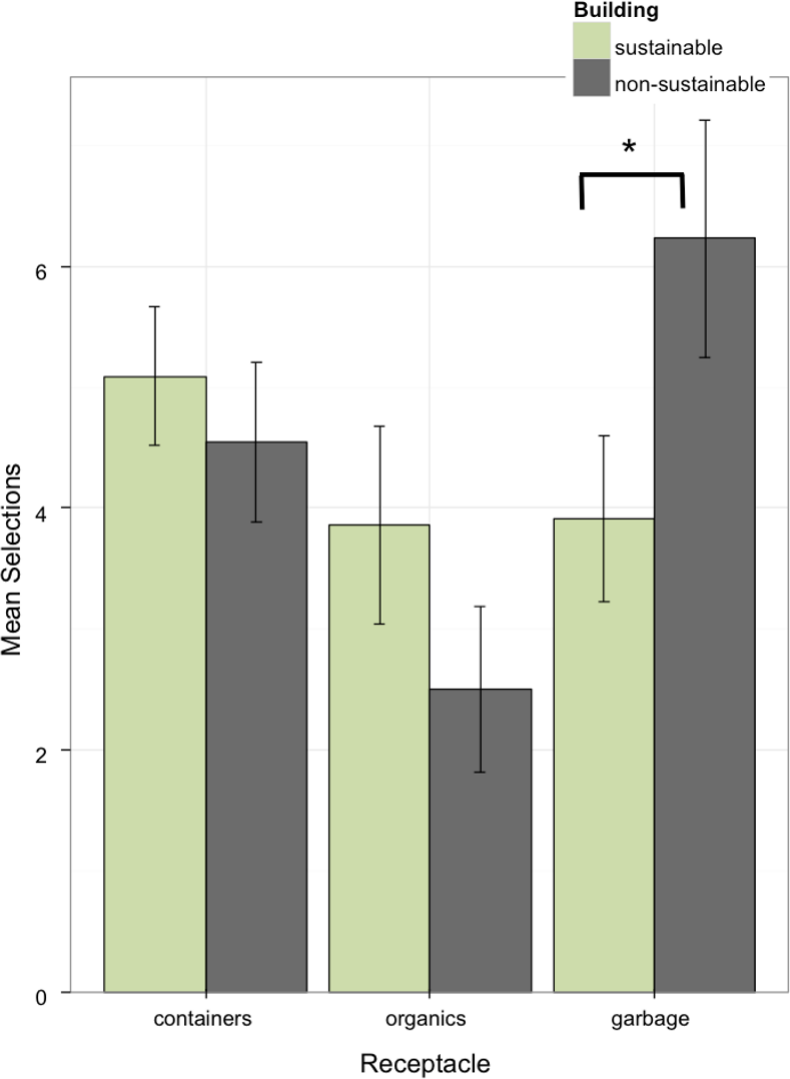
The number of times containers, organics, and garbage receptacles were selected in a sustainable building versus a non-sustainable building. Participants in the sustainable building condition used the garbage receptacle less than those in the non-sustainable building. As noted by the asterisk this difference was significant between the buildings.

A 2 (building) x 3 (receptacle type) between-within ANOVA showed an interaction between building and type, *F(2*, *84)* = 4.27, p = .017, η^2^ = .051. As illustrated in Fig 4, participants in the sustainable building were less likely to select the garbage receptacle than participants in the non-sustainable building, *F(1*, *84)* = 6.13, *p* = .015, η^2^ = .037. The organics and containers receptacles showed no significant difference between buildings, *F(1*, *84)* = 2.12, *p* = .15, η^2^ = .013, and *F(1*, *84)* < 1, η^2^ = .002, respectively. Viewing Fig 4 we see that in contrast to performance in the non-sustainable building participants in the sustainable building shifted their responses away from the garbage and towards the containers and organics receptacle.

### Accuracy

**Fig 5:**
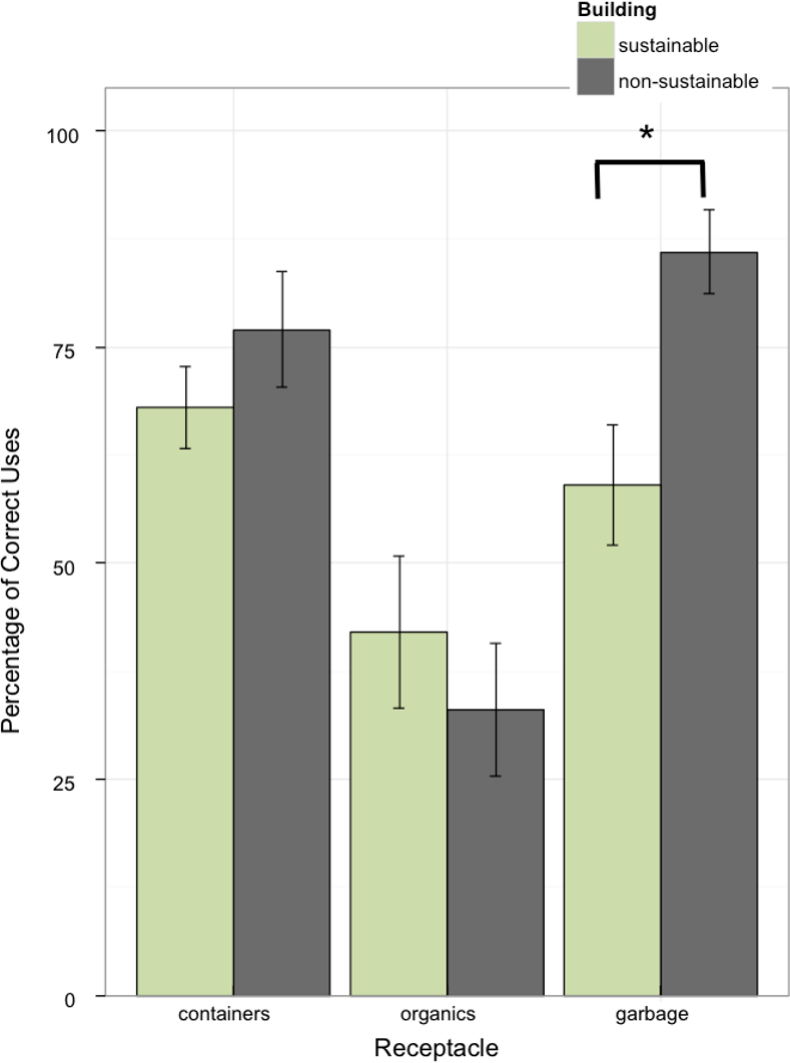
Average percentage of correct uses of waste receptacles across receptacle type and building type. A greater percentage of items were accurately placed in the garbage receptacle in the non-sustainable building.

To analyze accuracy, we determined what percentage of the six items belonging in each receptacle was disposed of correctly. A 2 (building) x 3 (receptacle type) between-within ANOVA revealed an interaction of building and receptacle type, *F*(2, 84) = 3.27, *p* = .043, η^2^ = .050. As illustrated in Fig 5, in the non-sustainable building, a greater percentage of items were accurately placed in the garbage receptacle, *F(1*, *84)* = 7.40, *p* = .0079, η^2^ = .057. There were no other differences between buildings for container or organic receptacles (both *F*s <1).

### Bin contents

The global bin contents are displayed in Table 2. Consistent with the above analyses indicating that people in the sustainable building were disinclined to dispose of items in the garbage, χ^2^ tests showed that the garbage receptacle received less garbage items in the sustainable building than in the non-sustainable building, χ^2^*(1)* = 6.41, *p* = .011, and the containers and the organics receptacles received more garbage items in the sustainable building than the non-sustainable building, χ^2^*(1)* = 7.71, *p* = .0055, and χ^2^*(1)* = 10, *p* = .0016, respectively.

**Table 2:** Number of items found in each receptacle type based on building condition. Bolded numbers represent significant differences based on χ^2^ tests (*p* < .05).

	Sustainable Building	Non-Sustainable Building
*Containers Receptacle*		
Container items	93	102
Compost items	6	6
Garbage items	**30**	**12**
*Organics Receptacle*		
Container items	5	3
Compost items	55.5	44
Garbage items	**10**	**0**
*Garbage Receptacle*		
Container items	10	16
Compost items	55.5	73
Garbage items	**78**	**113**

### Time spent at receptacles

We coded how much time participants spent at the receptacles in the current study, and how much time patrons spent at the receptacles in the Wu et al. [28] study. Videos of patrons from the observational study were randomly selected (25 from CIRS and 24 from the SUB). Coding was again conducted by two research assistants, one blind to the study. Coding reliability was high as shown by Cronbach’s alpha, α = .99.

**Fig 6:**
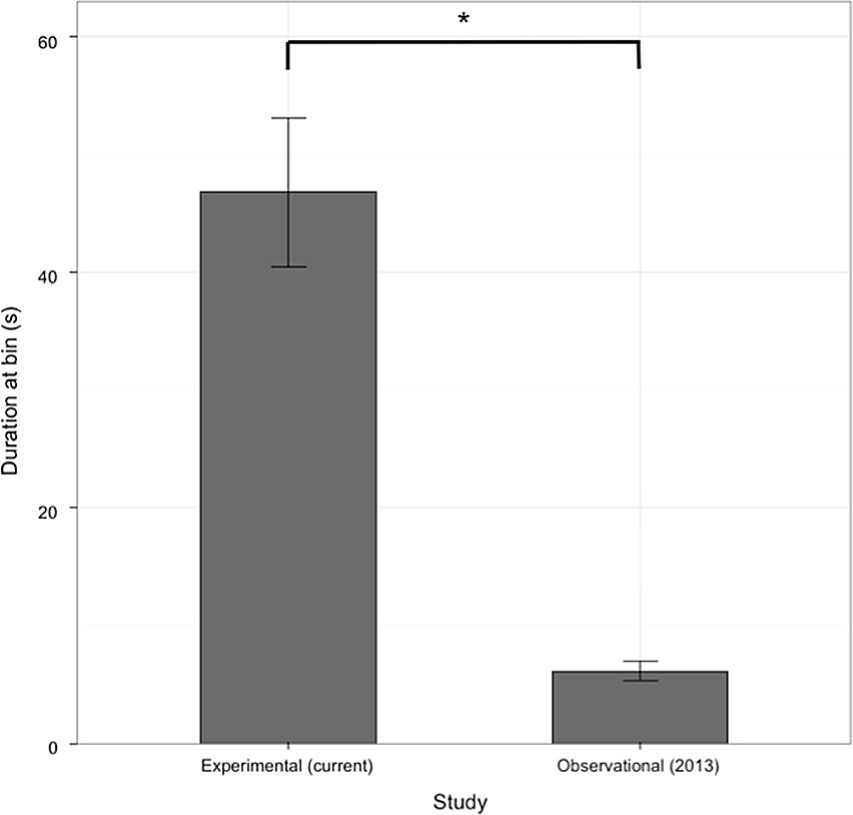
The duration of time spent at the receptacles in the current experimental study and in our observational study [26]. Participants spent significantly more time at receptacles in the current study compared to patrons in Wu et al. [28].

As illustrated in Fig 6, we found that participants spent significantly more time at the receptacles than patrons recorded in the Wu et al. [28] study, with participants spending on average 46.8 seconds at the receptacles in the current study compared to 6.1 seconds in the Wu et al. [28] data: *Welch’s t(44*.*3)* = 6.37, *p* < .001. There were no significant differences between the duration participants spent at the receptacles between the buildings in the current study: 52.5 seconds in the sustainable building versus 41.0 seconds in the non-sustainable building, *t(42)* = .91, *p* = .37. There was also no difference between the duration participants spent at receptacles between buildings in the observational data set: 7.5 seconds in the sustainable building versus 4.7 seconds in the non-sustainable building, *t(42)* = 1.84, *p* = .072.

## Discussion

Following our previous observational study [28], in the present study we randomly assigned participants to a sustainable building or a non-sustainable building to examine systematically how building context may influence recycling behavior. By using an experimental design we controlled for variation in the infrastructure and arrangement of the receptacle stations, individual differences, and changes in the goal-frames between the two buildings. Thus if the buildings differentially impacted recycling behavior, it would be by acting on motivational factors. Such normative influences should have a strong impact on low barrier, low reward behaviors as predicted by Schultz [18].

Our results converge on the conclusion that the atmospherics of a building can be a driver of recycling behavior and can do so by acting on motivational factors. Specifically, we found that participants in the sustainable building shifted their disposal strategies by decreasing the use of the garbage receptacle, while increasing the use of the container and organics receptacles.

This conclusion is supported by three lines of converging evidence. Our selection analysis found that participants in the sustainable building chose the garbage receptacle significantly less than participants in the non-sustainable building. And our accuracy and content analyses showed that participants in the sustainable building were so predisposed to avoid the garbage that garbage items were reassigned erroneously to the container and organics receptacles. It appears clear that sustainable building participants wanted to use the garbage receptacle less.

The fact that while in an experimentally controlled setting participants shifted their bin selection choices in a sustainable building compared to a non-sustainable building is supportive of Schultz [18]’s prediction that normative influences should have a strong impact on low barrier, low reward behaviors. However, our results cannot be interpreted as ruling out other ways in which a building might also impact pro-environmental behavior, as theorized by Steg and Vlek [19]. One’s built environment may very well influence other social-motivational factors, like social modeling or conformity pressures [34], and could be mediated by personal values or attitudes, change one’s goal-frames, or influence behavior directly through infrastructure changes.

The theory of planned behavior separates intent and behavior into distinct stages. Our results reveal the importance of this separation, and the need for both informational and motivational interventions [35]. While participants in the sustainable building selected the garbage receptacle less they were actually making more errors doing so, as shown in the accuracy analysis. This was also evidenced in the bin content analysis, where more garbage items ended up in the container and organics receptacles in the sustainable building. Therefore, while participants in the sustainable building condition inhibited their default selection of garbage with the intention of placing more items in organics or containers, they appear to have done so without thinking clearly or knowing about where things should ultimately be deposited.

The distinction between intention and behavior that our study highlights raises an intriguing possibility that may serve to advance our understanding of habitual behavior, an often forgotten aspect of pro-environmental behavior [15]. Sorting out waste is relatively unique in that it is performed routinely and there may be built-up habits or schemas surrounding the behavior. For example, the results in this study and in Wu et al. [28] suggest that garbage tends to be the default bin that is used. Changing waste disposal behavior first requires active processes to break acquired norms in order to conform to new ones [36]. Verplanken and Wood [37] suggest that combining contextual changes that eliminate the triggering cues for the habit with other interventions that target motivational factors like norms can be an effective method to break habits. Our results suggest that buildings conveying an atmosphere of sustainability may serve to do both effectively. Future research should focus on whether other habitual behaviors can be broken with changes in the built environment.

### More time spent at receptacles does not result in better waste disposal

Our study also raises another new and intriguing conclusion–that the duration one spends at the receptacles need not predict performance quality. This conclusion is supported by our results showing that 1) participants in the experimental study spent significantly more time at the receptacles than did patrons in the natural setting investigated by Wu et al. [28], and 2) that in both studies, building type had no impact on the duration participants spent at the receptacles, yet building type had a considerable impact on disposal behavior.

The increase in time spent at the receptacles in the present study compared to the observational study of Wu et al. [28] indicates that, as predicted, behavior changes as a function of the experimental context itself. Possible reasons include participants in the present study being under less time pressure, participants acting more deliberately in experimental settings, and/or the fact that they were aware of the presence of the experimenter and dwelled at the receptacles as an overt pro-social signal. This last possibility is particularly intriguing. While it is well known that social presence can increase pro-social behavior [38], our finding may suggest that the nature of that presence may be important as well. In an experimental situation, where the presence is one of potential authority and may be judging an individual, the participant will be more motivated to act pro-socially (i.e., motivated by their image: [39]). In a cafeteria setting, where there are a large number of people, an individual can stay anonymous and the motivation to act pro-socially might be much weaker.

## Future directions

As noted previously, our results also suggest that the duration one spends at the receptacles does not predict the quality of decision making at the receptacles. We have offered several explanations for this result, and future studies may explore those explanations further by manipulating the time participants have to make a decision and the presence of an authority. However, whatever the underlying mechanism is, the fact that participants did act significantly different in their waste disposal behavior in a controlled experimental setting compared to a natural setting should be noted by researchers seeking to study natural behaviors. It underscores that there are disadvantages when studying these behaviors in strictly controlled experimental settings. Low sample size, demand characteristics, and general differences in how participants behave create a need to couple such experiments with naturalistic observational studies [40].

Our main results suggest that the atmospherics of a sustainable building can influence waste disposal behavior through motivational mechanisms. Earlier, we noted however that these results do not exclude other mechanisms through which a building may influence behavior as predicted by Steg and Vlek [19]. Indeed, the difference in accuracy between the current study and the observational results in Wu et al. [28] suggests that in a natural situation, one or more of factors suggested by Steg and Vlek [19] may be in play. We suspect that accuracy may be lower in the current study because of the variety of items participants were disposing of, whereas in a cafeteria setting patrons are routinely throwing out the same few items. But future research will most certainly have to systematically isolate and investigate how a building might act through these other mechanisms, and what mechanism or designs can most effectively increase accuracy. We have already begun to move in this direction by investigating the design of waste disposal signage.

Moreover, though the present data are convergent with the conclusion that a building can impact pro-environmental behavior through normative influences, this does mean that this effect is being driven by only one norm. That is, the building may also be changing the saliency of a number of other norms [8]. For example, there may be norms activated by the “newness” or cleanliness of a building in addition to than the “greenness” of it [41]. Future studies could isolate this effect by testing participants in a new non-sustainable building versus an old non-sustainable building, and compare the results with those from a new sustainable building versus an old sustainable building. Other atmospherics or design features may very well impact waste disposal behavior in different ways. The current study is just the first step on what looks to be a very promising line of research.

## Conclusion

The current study follows on the observational study in Wu et al. [28] by using a controlled experimental research design. We find that participants select the garbage receptacle less in a sustainable building compared to a non-sustainable building, but they do not necessarily perform more accurately. Our study supports the notion that the built environment can influence low barrier, low reward behaviors like recycling. Such influences may be important to break old habits like choosing the garbage as the default receptacle. However, other mechanisms may remain important in achieving a desired behavior. While design can influence people to behave more pro-socially, it is not the final answer to improve recycling performance. Our study points to the power of design in promoting pro-environmental motivations, but also exposes the limits of turning such motivations to a specific desired outcome.
